# Characterization of the Nuclear Import Mechanism of the CCAAT-Regulatory Subunit Php4

**DOI:** 10.1371/journal.pone.0110721

**Published:** 2014-10-17

**Authors:** Md. Gulam Musawwir Khan, Jean-François Jacques, Jude Beaudoin, Simon Labbé

**Affiliations:** Département de Biochimie, Faculté de Médecine et des Sciences de la Santé, Université de Sherbrooke, Sherbrooke, Québec, Canada; Institut de Recherches Cliniques de Montréal (IRCM), Canada

## Abstract

Php4 is a nucleo-cytoplasmic shuttling protein that accumulates in the nucleus during iron deficiency. When present in the nucleus, Php4 associates with the CCAAT-binding protein complex and represses genes encoding iron-using proteins. Here, we show that nuclear import of Php4 is independent of the other subunits of the CCAAT-binding complex. Php4 nuclear import relies on two functionally independent nuclear localization sequences (NLSs) that are located between amino acid residues 171 to 174 (KRIR) and 234 to 240 (KSVKRVR). Specific substitutions of basic amino acid residues to alanines within these sequences are sufficient to abrogate nuclear targeting of Php4. The two NLSs are biologically redundant and are sufficient to target a heterologous reporter protein to the nucleus. Under low-iron conditions, a functional GFP-Php4 protein is only partly targeted to the nucleus in *imp1Δ* and *sal3Δ* mutant cells. We further found that cells expressing a temperature-sensitive mutation in *cut15* exhibit increased cytosolic accumulation of Php4 at the nonpermissive temperature. Further analysis by pull-down experiments revealed that Php4 is a cargo of the karyopherins Imp1, Cut15 and Sal3. Collectively, these results indicate that Php4 can be bound by distinct karyopherins, connecting it into more than one nuclear import pathway.

## Introduction

In eukaryotic cells, the nucleus is a membrane-enclosed organelle that physically separates genetic material and transcriptional machinery from cytoplasm. Although proteins are translated in the cytoplasm, several of them play important roles in the nucleus. In order to accomplish their cellular function, they must be imported into the nucleus. The way that proteins can be transported into and out of the nucleus is through large protein assemblies denoted nuclear pore complexes (NPCs) [1]. Although some proteins smaller than ∼40–0 kDa can passively diffuse through NPCs, most of the proteins with functions in the nucleus are actively transported by specific soluble carrier proteins called karyopherins (Kaps) [2], [3]. The orientation of transport through NPCs is determined by short signal sequences within proteins or cargoes. The nuclear localization signal (NLS) triggers proteins into the nucleus, whereas the nuclear export signal (NES) fosters the transport of proteins into the cytoplasm [4]. Kaps are responsible for the vast majority of protein flow through NPCs. Kaps are classified in two families: Kap α (also known as importin α) and Kap β (also known as importin β) [5]. Kap α is an adaptor protein that recognizes two classes of NLSs, which are also called classical NLSs [6]. One class, denoted monopartite NLS, is composed of a single cluster of basic amino acid residues, whereas the second class, termed bipartite NLS, possesses two clusters of basic amino acid residues separated by a 10–2-amino acid spacer. Furthermore, there are two types of monopartite NLSs. The first type has at least four consecutive basic amino acid residues in its primary structure, whereas the second type possesses the degenerate consensus sequence of K(K/R)X(K/R) [6]. To be transported in the nucleus, a protein containing a classical NLS is recognized by a Kap α. Subsequently, a Kap β1 binds the Kap α-cargo-complex to mediate its transport across NPCs. Kap β1 interacts with both Kap α-cargo-complex and NPC proteins (nucleoporins), thereby targeting the cargo to the NPC for its translocation into the nucleus [6], [7]. Numerous proteins contain nonclassical NLSs. These proteins bind directly and specifically to different Kap β1 homologs that constitute the Kap β family [2]. Kap β1 is unique among the Kap β family in its use of Kap α as an adaptor protein. Other members of the Kap β family bind their substrates directly [2]. The dissociation of Kap β-cargo complexes is under the control of the GTPase Ran. Inside the nucleus, Ran-nucleotide guanine triphosphate (GTP) binds to Kap β-cargo complexes, resulting in the dissociation and release of cargoes into the nucleus [8].

In the fission yeast *Schizosaccharomyces pombe*, Imp1 and Cut15 are two members of the Kap α family [9]. In the case of Kap βs, twelve candidates have been annotated from the *S. pombe* Genome Project [10]. Although the majority of them have not yet been characterized, Kap95 is predicted to be the ortholog of *S. cerevisiae* Kap95, which is a Kap β1 [2], [11]. Recent studies have also shown that Kap104 is a Kap β2-type receptor, which mediates nuclear import of proline-tyrosine (PY)-NLS cargoes [12]. Unlike classical NLSs, PY-NLS consensus sequence corresponds to [basic/hydrophobic]-X_n_-[R/H/K]-X_2–5_-PY [12], [13].

Iron-regulatory transcription factors play fundamental roles by controlling expression of multiple genes encoding proteins involved in iron homeostasis. In the model organism *S. pombe*, regulation of iron homeostasis is mainly controlled by two iron-responsive proteins, the GATA-binding transcription factor Fep1 and the CCAAT-regulatory subunit Php4 [14]. When iron levels exceed those needed by the cells, Fep1 binds to GATA-type *cis*-acting elements and represses the expression of a number of genes involved in iron transport and intracellular iron utilization [15], [16]. In contrast, Fep1 is unable to bind chromatin in response to iron deficiency [17]. This situation leads to transcriptional activation of the Fep1 regulon, which includes the *php4^+^* gene [18], [19]. During iron starvation, Php4 is synthesized and coordinates the iron-sparing response by repressing many genes encoding iron-using proteins [19]. At the molecular level, Php4 regulates its target genes by recognition of the CCAAT-binding complex which is constituted of Php2, Php3 and Php5. The Php2/3/5 heterotrimer binds CCAAT *cis*-acting elements whereas Php4 lacks DNA-binding activity. Php4 is responsible for the ability of the Php complex to repress transcription as a consequence of its association with the heteromeric complex [18], [19]. As for Fep1 orthologs, Php4-like proteins are widely distributed in other fungal species. *Saccharomyces* species is the only group that lacks Php4 and Fep1 orthologs [20].

Studies have shown that the monothiol glutaredoxin Grx4 is a binding partner of Php4 and that it plays an essential role in inhibiting Php4 function when cells undergo a shift from iron-limiting to iron-replete conditions [21], [22]. Under conditions of iron abundance, Php4 is exported from the nucleus to the cytoplasm. The nuclear export of Php4 requires both exportin Crm1 and Grx4 [21]. Consistently, disruption of the *grx4^+^* gene (*grx4Δ*) results in Php4 being constitutively active and invariably located in the nucleus. Although the mechanism by which Grx4 communicates the high concentrations of iron to Php4 remains unclear, deletion mapping analysis revealed that the thioredoxin (TRX) domain of Grx4 interacts strongly and constitutively with Php4 [22]. Further analysis has revealed that, in response to iron repletion, the glutaredoxin (GRX) domain of Grx4 associates with Php4. A putative mechanism for Grx4-mediated inhibition of Php4 function would be that the Php4-GRX domain iron-dependent association disrupts the Php4/Php2/Php3/Php5 heteromeric complex, leading to Php4 release and its subsequent export from the nucleus to the cytoplasm by Crm1.

Exported Php4 is observed in the cytosol. However, when external growth conditions change and cells are exposed to iron-poor conditions, it follows that nuclear localization of Php4 should be re-established via its import to the nucleus. To address this issue, we have characterized the mechanism of cytosolic-to-nuclear import of Php4. In response to iron deficiency, nuclear import of Php4 occurred and deletion of *php2^+^*, *php3^+^* and *php5^+^* (*php2Δ php3Δ php5Δ*) did not cause any defects in its nuclear localization. Protein function analysis identified two independent and biologically redundant NLSs within Php4. Each NLSs was sufficient to target an unrelated reporter protein to the nucleus. Disruption of *imp1Δ* or *sal3Δ* gene caused GFP-Php4 to partly mislocalize to the cytoplasm under low-iron conditions. Similarly, in cells containing a temperature-sensitive mutation of *cut15*, GFP-Php4 was mistargeted to the cytoplasm at the nonpermissive temperature. Further analysis by pull-down experiments showed that Php4 interacted with Imp1, Cut15 and Sal3 in *S. pombe*. Collectively, our findings show that Php4 possesses two nuclear targeting sequences that are used by different Kaps for its nuclear import in response to iron starvation.

## Materials and Methods

### Strains and growth media

*S. pombe* strains used in this study are listed in Table 1. Cells were grown in yeast extract medium plus supplements (YES) containing 0.5% yeast extract, 3% glucose, and 225 mg/l of adenine, histidine, leucine, uracil and lysine. Strains for which plasmid transformation was required were grown in synthetic Edinburgh minimal medium (EMM) lacking specific amino acids required for plasmid selection and maintenance [23]. Cells constitutively expressing a *GFP-php4^+^* allele were seeded to an *A_600_* of 0.2, grown to mid-logarithmic phase (*A_600_* of 0.5) and then treated with either 2,2′-dipyridyl (Dip, 250 µM) or FeCl_3_ (100 µM), or were left untreated for 3 h, unless otherwise stated. When the wild-type or mutant *php4* alleles were expressed under the control of the *nmt1^+^* promoter, induction of transcription was initiated by removal of thiamine to cells grown to an *A_600_* of 0.2. After 12 h of induction, cells were incubated with Dip (250 µM) or FeCl_3_ (100 µM) for 3 h. In contrast, to prevent expression of *php4* alleles, cells were grown in the presence of thiamine (15 µM or 45 µM), unless otherwise indicated. In the case of *cut15–85^ts^* cells expressing a thermolabile Cut15, cells were grown at the permissive temperature (25°C) to an *A_600_* of ∼0.4. The cells were then shifted to 36°C for 1 h and then further incubated at 36°C in the presence of Dip (250 µM) or FeCl_3_ (100 µM) for an additional 3 h.

**Table 1:** *S. pombe* strain genotypes.

Strain	Genotype	Source orreference
FY435	h^+^ his7–366 leu1–32 ura4-Δ18 ade6-M210	[48]
AMY17	h^+^ his7–366 leu1–32 ura4-Δ18 ade6-M210 php4Δ::loxP	[21]
GKY1	h^+^ his7–366 leu1–32 ura4-Δ18 ade6-M210 php4Δ::loxP php2Δ::loxP php3Δ::loxP php5Δ::loxP	This study
GKY2	h^+^ his7::loxP leu1–32 ura4-Δ18 ade6-M210 kap104Δ::natMX6 php4Δ::KAN^r^	This study
GKY3	h^+^ his7–366 leu1–32 ura4-Δ18 ade6-M210 imp1Δ::loxP php4Δ::KAN^r^	This study
GKY4	h^+^ his7–366 leu1–32 ura4-Δ18 ade6-M210 sal3Δ::loxP php4Δ::KAN^r^	This study
GKY5	h^+^ his7–366 leu1–32 ura4-Δ18 ade6-M210 imp1Δ::loxP sal3Δ::loxP php4Δ::KAN^r^	This study
GKY6	h^+^ his7Δ::loxP leu1–32 ura4Δ::loxP ade6Δ::loxP php4Δ::KAN^r^	This study
GKY7	h^+^ his7Δ::loxP leu1–32 ura4Δ::loxP ade6Δ::loxP cut15–85 php4Δ::KAN^r^	This study
GKY8	h^+^ his7–366 leu1–32 ura4-Δ18 ade6-M210 php4Δ::loxP imp1^+^-TAP::KAN^r^	This study
GKY9	h^+^ his7–366 leu1–32 ura4-Δ18 ade6-M210 php4Δ::loxP sal3^+^-TAP::KAN^r^	This study
GKY10	h^+^ his7–366 leu1–32 ura4-Δ18 ade6-M210 php4Δ::loxP cut15^+^-TAP::KAN^r^	This study

### Plasmids

pJK-194*prom*php4^+^*-*GFP-php4^+^* plasmid has been described previously [21]. Plasmids pJKGFP-^1^Php4^88^, pJKGFP-^1^Php4^144^, pJKGFP-^1^Php4^179^, pJKGFP-^1^Php4^218^, pJKGFP-^152^Php4^295^, pJKGFP-^188^Php4^295^, pJKGFP-^219^Php4^295^, and pJKGFP-^245^Php4^295^ were created by cloning different truncated versions of the *php4^+^* gene into pJK-194*prom*php4^+^*-*GFP-php4^+^*. Different lengths of *php4^+^* were generated by PCR using primers that contained SalI and Asp718 restriction sites at their ends. After amplification, purified DNA fragments were digested with these two enzymes and then swapped into the corresponding sites of pJK-194*prom*php4^+^*-*GFP-php4^+^*, generating a series of plasmids bearing deletions within different regions of *php4^+^*. To create *php4* mutant alleles K171A/R172A/I173/R174A, K214A/I215/R216A/K217A/R218A, and K234A/S235/V236/K237A/R238A/V239A/R240A, the plasmid pJK-194*prom*php4^+^*-*GFP-php4^+^* was used in conjunction with the overlap extension method [24]. Primers were designed to ensure the presence of nucleotide substitutions that gave rise to the above-mentioned mutations. Using two additional oligonucleotides corresponding to the start and stop codons of the ORF of *php4^+^*, overlap extension PCR allowed generation of *php4-K171A/R172A/I173/R174A, php4-K214A/I215/R216A/K217A/R218A, and php4-K234A/S235/V236/K237A/R238A/V239A/R240A* alleles. These mutant alleles were used to replace the equivalent wild-type *php4^+^* DNA segment in pJK-194*prom*php4^+^*-*GFP-php4^+^*. Similarly, overlap extension PCR was used to generate additional *php4* mutants that included different combinations of *K171A/R172A/I173/R174A* mutations with *K214A/I215/R216A/K217A/R218A* or/and *K234A/S235/V236/K237A/R238A/V239A/R240A* mutations. Plasmid pSP-1178nmt-GST-GFP [25] was digested with SpeI and SacI restriction enzymes and used to join annealed synthetic DNA fragments encoding wild-type versions of SV40 NLS and Pap1 NES [26]–[28]. Wild-type *php4^+^* coding regions corresponding to amino acid residues 160–190, 188–224, and 219–246 were isolated by PCR and cloned downstream of and in-frame to *GST-GFP* fusion genes, generating plasmids pSP-1178nmt-GST-GFP-^160^Php4^190^, pSP-1178nmt-GST-GFP-^188^Php4^224^, and pSP-1178nmt-GST-GFP-^219^Php4^246^, respectively. Similarly, these *php4^+^* coding regions (amino acid residues 160–190, 188–224, and 219–246) were amplified from plasmids pJK-194*prom*php4^+^*-*GFP-php4-K171A/R172A/I173/R174A,* pJK-194*prom*php4^+^*-*GFP-php4-K214A/I215/R216A/K217A/R218A,* and pJK-194*prom*php4^+^*-*GFP-php4-K234A/S235/V236/K237A/R238A/V239A/R240A* to create plasmids pSP-1178nmt-GST-GFP-mutant^160^Php4^190^, pSP-1178nmt-GST-GFP-mutant^188^Php4^224^, and pSP-1178nmt-GST-GFP-mutant^219^Php4^246^, respectively. The wild-type *php4^+^* coding region corresponding to amino acid residues 160–246 was amplified by PCR using primers designed to generate SpeI and SacI sites at each extremity of the PCR product. The DNA fragment was inserted into the corresponding sites of pSP-1178nmt-GST-GFP. The resulting plasmid, named pSP-1178nmt-GST-GFP-^160^Php4^246^, was subsequently used to create three additional plasmids harboring *K171A/R172A/I173/R174A*, *K234A/S235/V236/K237A/R238A/V239A/R240A* or *K171A/R172A/I173/R174A*/*K234A/S235/V236/K237A/R238A/V239A/R240A* substitutions.

### RNase protection analysis

Total RNA was extracted using a hot phenol method as described previously [29]. In the case of RNase protection assays, RNA (15 μg per reaction) was hybridized and digested with RNase T1 as described previously [19]. Riboprobes derived from plasmids pSK*isa1^+^* and pSK*act1^+^* [18] were used to detect *isa1^+^* and *act1^+^* transcripts, respectively. Plasmids were linearized with BamHI for subsequent antisense RNA labeling with [α-^32^P]UTP and T7 RNA polymerase. *act1^+^* mRNA was probed as an internal control for normalization during quantification of RNase protection products.

### Fluorescence microscopy analysis

Fluorescence microscopy was performed as described previously [30]. Both fluorescence and differential interference contrast images (Nomarski) of cells were obtained using a Nikon Eclipse E800 epifluorescent microscope (Nikon, Melville, NY) equipped with a Hamamatsu ORCA-ER digital cooled camera (Hamamatsu, Bridgewater, NJ). Samples were analyzed using a 1,000X magnification with the following filters: 520 to 550 nm (YFP), 465 to 495 nm (GFP), and 340 to 380 nm (Hoechst 33342). Cell fields shown in this study represent a minimum of five independent experiments.

### TAP pull-down experiments

For pull-down experiments, we created *php4Δ* null strains in which the TAP coding sequence was integrated at the chromosomal locus of *imp1^+^*, *cut15^+^*, or *sal3^+^*. These integrations were performed using a PCR-based gene fusion approach as described previously [31], except that pFA6a-kanMX6-CTAP2 [32] was used to amplify the TAP coding sequence. The method allowed homologous integration of TAP at the chromosomal locus of *imp1^+^*, *cut15^+^*, or *sal3^+^*, thereby replacing wild-type allele by *imp1^+^-TAP*, *cut15^+^-TAP* or *sal3^+^-TAP* allele. To determine whether Php4 interacted with Imp1, Cut15 or Sal3 in *S. pombe*, *php4Δ imp1^+^-TAP*, *php4Δ cut15^+^-TAP*, or *php4Δ sal3^+^-TAP* cells were transformed with pBPade6^+^-nmt41x-GFP-php4^+^. The cells were grown to mid-logarithmic phase in a thiamine-free medium and then treated with Dip (250 µM) for 3 h. Total cell lysates were prepared as described previously [33], except that PMSF (1 mM) was directly added to cell cultures 10 min before cell lysis. Preparation of IgG-Sepharose 6 Fast-Flow beads (GE Healthcare) and coupling of proteins to beads were carried out as described previously [33]. After end-over-end mixing for 30 to 60 min at 4°C, the beads were washed four times with lysis buffer (1 ml each time) and then transferred to a fresh microtube prior to a final wash. The immunoprecipitates were resuspended in sodium dodecyl sulfate loading buffer (60 µl), heated for 5 min at 95°C and proteins resolved by electrophoresis on 9% sodium dodecyl sulfate-polyacrylamide gels. The following antibodies were used for Western blotting analysis of Imp1-TAP, Cut15-TAP, Sal3-TAP, GFP-Php4 and α-tubulin: polyclonal anti-mouse IgG antibody (1∶500) (ICN Biomedicals); monoclonal anti-GFP antibody B-2 (1∶500) (Santa Cruz Biotechnology) and monoclonal anti-α-tubulin antibody (1∶5000) (clone B-5-1-2; Sigma-Aldrich). Following incubation with primary antibodies, membranes were washed and incubated with the appropriate horseradish peroxidase-conjugated secondary antibodies (1∶5000) (Amersham Biosciences), developed with enhanced chemiluminescence (ECL) reagents (Amersham Biosciences) and visualized by chemiluminescence.

## Results

### Localization of Php4 to the nucleus in a Php2/Php3/Php5-independent manner under low-iron conditions

**Figure 1:**
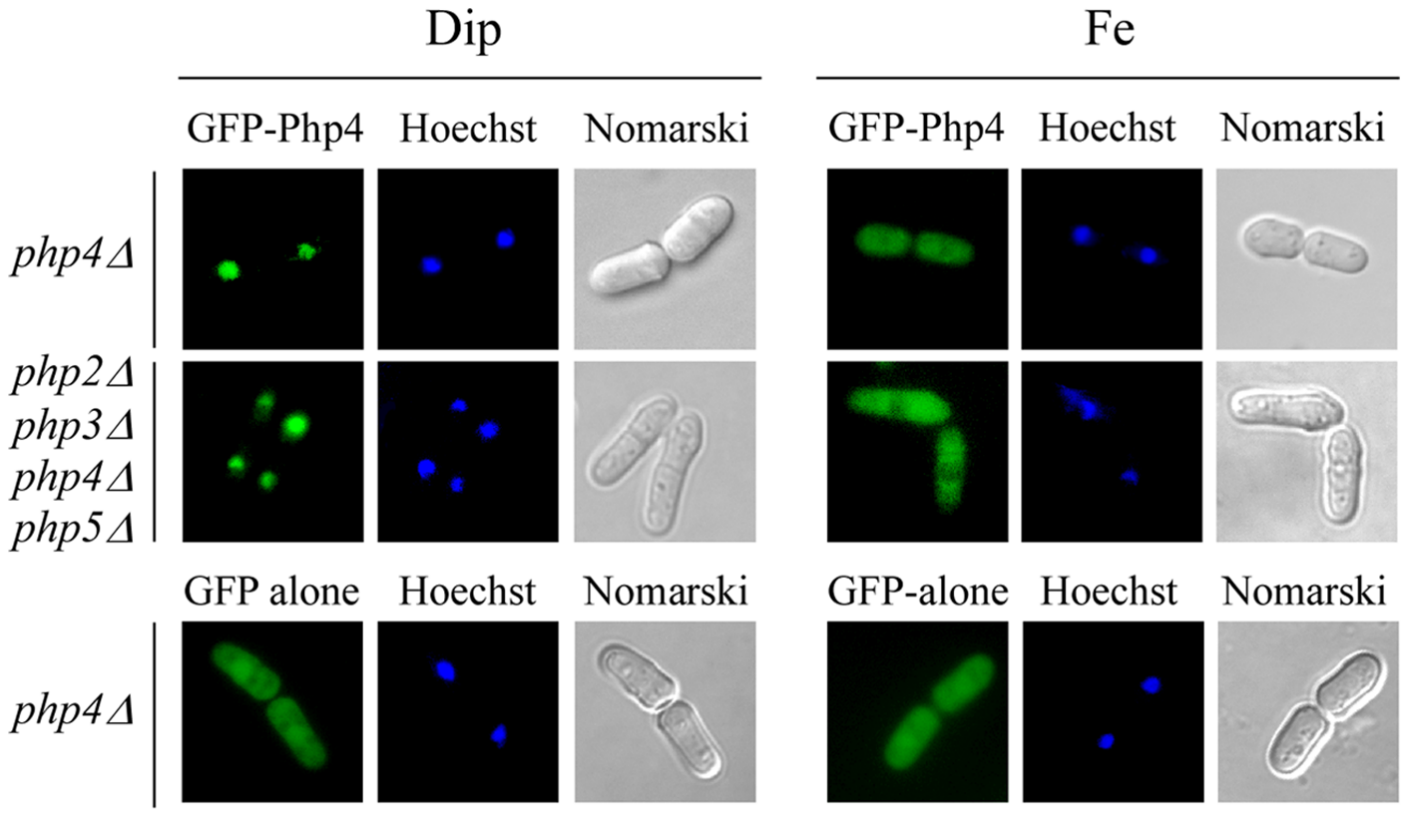
Iron*-*regulated nucleo-cytoplasmic trafficking of *Php4* is independent of *Php2, Php3* and *Php5* proteins. *php4Δ* or *php2Δ php3Δ php4Δ php5Δ* mutant cells were transformed with an integrative vector expressing GFP alone or a *GFP-php4^+^* allele under the control of a GATA-less *php4^+^* promoter. Transformed cells were treated with either Dip (250 μM) or FeCl_3_ (Fe) (100 μM) for 3 h. Nuclear DNA was visualized by Hoechst staining whereas Nomarski optics (Nomarski) was used to reveal cell morphology. For simplicity, only *php4Δ* cells transformed with GFP alone are shown because fluorescent images of *php2Δ php3Δ php4Δ php5Δ* cells were identical. The results shown are representative of five independent experiments.

As we have previously shown, functional GFP-Php4 localized in the cytoplasm of cells under iron-sufficient conditions (Fig. 1) [21]. Conversely, GFP-Php4 accumulated in the nucleus when cells underwent a transition from iron-sufficient to iron-limiting conditions (Fig. 1) [21]. To further investigate the mechanism by which GFP-Php4 was imported in the nucleus, we tested whether Php2, Php3, and Php5 were required for its nuclear accumulation in response to iron starvation. To perform these experiments, *php4Δ* and *php2Δ php3Δ php4Δ php5Δ* mutant strains were transformed with an integrative plasmid harboring a *GFP-php4^+^* allele constitutively expressed from a GATA-less *php4^+^* promoter. Cells expressing GFP-Php4 were grown under basal conditions to mid-logarithmic phase and then treated with the iron chelator Dip or with FeCl_3_ for 3 h. Results showed that in the presence of Dip, GFP-Php4 accumulated in the nucleus of both *php4Δ* and *php2Δ php3Δ php4Δ php5Δ* mutant strains (Fig. 1). In contrast, when these strains were treated with FeCl_3_, GFP-Php4 was observed primarily in the cytoplasm (Fig. 1). As we have previously observed, GFP alone displayed a pancellular-fluorescence pattern, regardless of cellular iron status (Fig. 1) [21]. Taken together, these results indicated that GFP-Php4 localizes to the nucleus in iron-starved cells in a Php2/Php3/Php5-independent manner. Conversely, in iron-replete cells, GFP-Php4 exhibits a distinct distribution pattern that is cytoplasmic.

### Mapping NLSs of Php4

**Figure 2:**
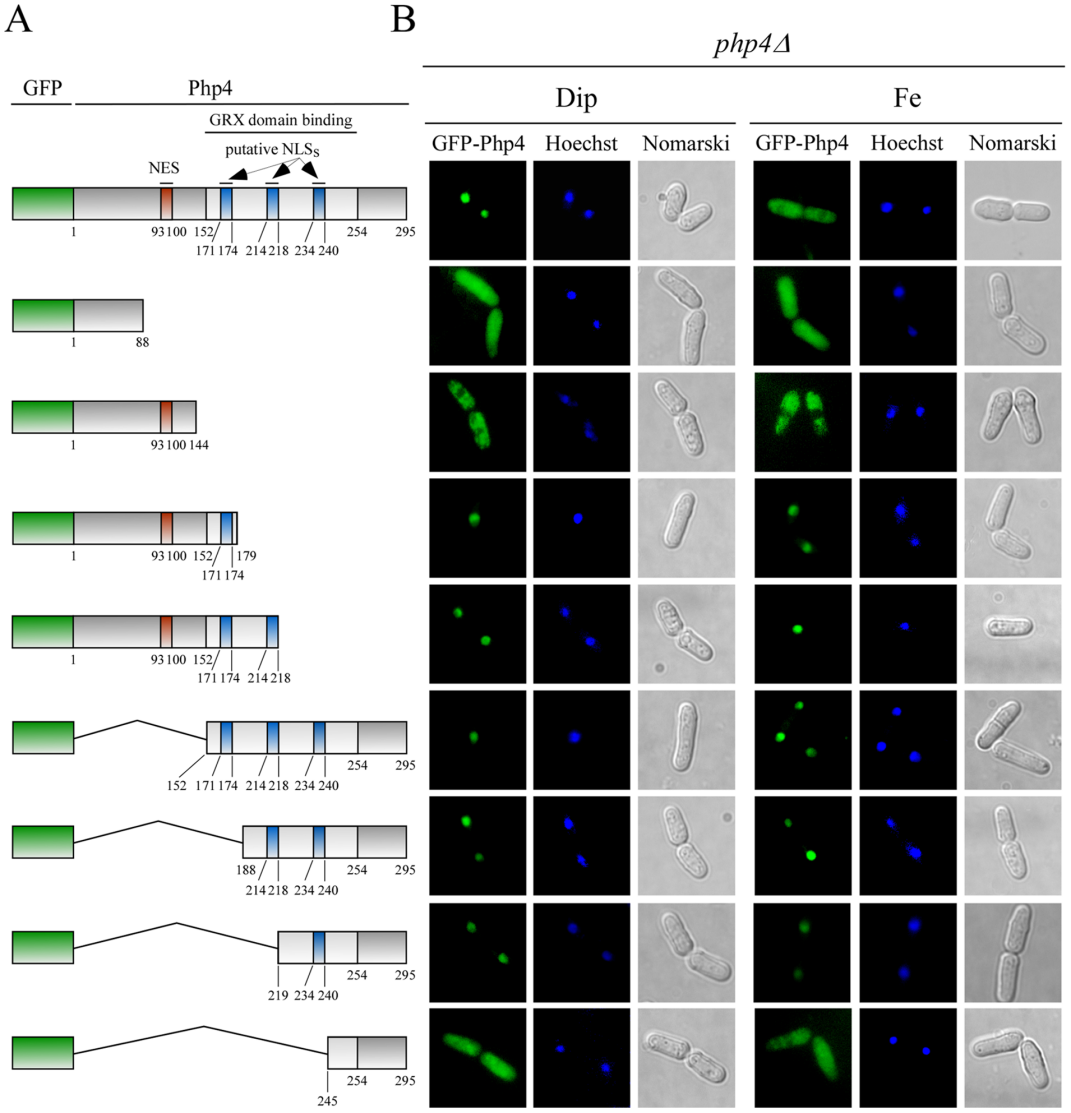
Distinct regions of *Php4* are required for its nuclear localization. *A*, Schematic representation of the GFP-Php4 fusion protein and different GFP-Php4 fusion derivatives. The red box indicates the nuclear export signal (NES) found in Php4 (residues 93–100). Blue boxes represent putative nuclear localization signals (NLSs) that were identified in Php4 (residues 171–174, residues 214–218 and residues 234–240). The segment encompassing residues 152–254 (light-grey box) is a C-terminal region of Php4 required for interaction with the GRX domain of Grx4, which is required for iron-mediated exportation of Php4. The green box represents the GFP coding sequence. The amino acid sequence numbers refer to the positions relative to the first amino acid of Php4. *B*, *php4Δ* cells expressing the indicated fusion alleles under the control of a GATA-less *php4^+^* promoter were incubated in the presence of Dip (250 μM) or FeCl_3_ (Fe) (100 μM). After 3 h, cells were examined by fluorescence microscopy to visualize GFP-Php4 and its different fusion derivatives. Hoechst staining revealed nuclear DNA whereas Nomarski optics was used to monitor cell morphology. The results shown are representative of five independent experiments.

To begin to characterize regions within Php4 responsible for nuclear localization, we created a series of N- and C-terminal deletions and fused GFP to the N terminus of each truncated protein (Fig. 2A). *php4Δ* cells expressing these truncated versions of GFP-Php4 were analyzed by fluorescence microscopy to identify which mutants localized to the nucleus. Truncated GFP-^1^Php4^88^, in which the last 207 amino acid residues of Php4 were deleted exhibited a pancellular-fluorescence pattern under both low and high iron concentrations (Fig. 2B), suggesting that GFP-^1^Php4^88^ was able to passively enter and exit the nucleus. In the case of GFP-^1^Php4^144^, results showed that it primarily accumulated in the cytoplasmic region of *php4Δ* cells (Fig. 2B). This finding was consistent with the presence of a NES encompassing amino acid residues 93–100 [21]. GFP-^1^Php4^179^ and GFP-^1^Php4^218^ were located in the nucleus under both iron-limiting and iron-replete conditions (Fig. 2B). Although their nuclear location was independent of the cellular iron status, these observations were consistent with the interpretation of the presence of at least one NLS encompassing a common minimal region composed of amino acid residues 144–179. One reason that may explain the absence of iron-mediated nuclear export of GFP-^1^Php4^179^ and GFP-^1^Php4^218^ is the fact that these chimeric proteins miss part of the C-terminal region (positions 152 to 254) of Php4. Previous structure-function studies have revealed that the association of the GRX domain of Grx4 and Php4 depends on the presence of this region (Php4 152–254) [22]. Furthermore, it is known that the GRX domain-Php4 association is required for the iron-mediated inhibition of Php4 that leads to its recruitment by Crm1 (via Php4 NES 93–100), and its subsequent export out of the nucleus to the cytoplasm [21]. Deletion of amino acid residues 1 to 151, 1 to 187, and 1 to 218 from the N-terminus to generate GFP-^152^Php4^295^, GFP-^188^Php4^295^, and GFP^219^Php4^295^ did not affect nuclear localization. Due to the absence of NES, GFP-^152^Php4^295^, GFP-^188^Php4^295^ and GFP^219^Php4^295^ were located exclusively in the nucleus, regardless of cellular iron status. However, further deletion of 26 amino acid residues in GFP^219^Php4^295^ to generate GFP^245^Php4^295^, nullified its ability to localize exclusively in the nucleus. Instead, GFP^245^Php4^295^ exhibited pancellular localization in iron-starved and iron-replete cells (Fig. 2B), revealing loss of signal to promote active entry of Php4 into the nucleus. Taken together, these results were consistent with the interpretation that regions of Php4 from amino acid residues 144 to 179, 188 to 245, and 219 to 245 are sufficient to mediate nuclear import and accumulation of Php4.

### Mutation of three predicted NLSs of Php4

**Figure 3:**
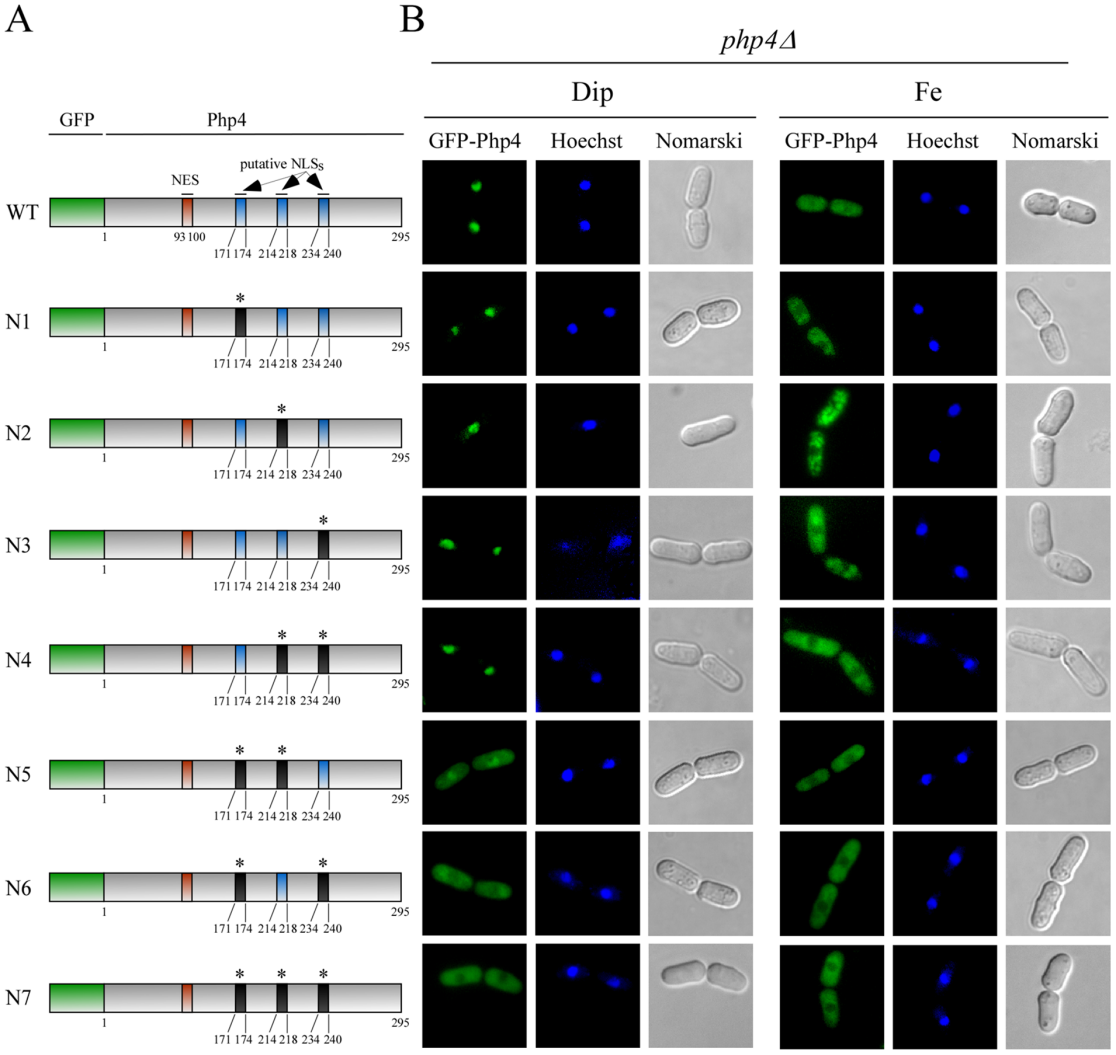
Two regions of *Php4* encompassing amino acid residues 171 to 174 and 234 to 240 are involved in targeting *Php4* to the nucleus. *A*, Schematic illustration of wild-type (WT) and mutant versions (N1 to N7) of GFP-Php4 fusion protein. Green, red and blue boxes represent GFP coding sequence, NES, and putative NLS, respectively. Black boxes (marked with an asterisk) indicate mutated NLS. The amino acid residues of Php4 are numbered relative to its initiator codon. *B*, Fluorescence microscopy was used to visualize cellular location of GFP-Php4 and its mutant derivatives that were expressed in *php4Δ* cells. When indicated, cultures were treated with Dip (250 μM) or FeCl_3_ (Fe) (100 μM) for 3 h. Cells were stained using Hoechst to visualize nuclear DNA, whereas Nomarski optics was used to monitor cell morphology. The results shown are representative of five independent experiments.

In light of these observations, we sought to identify amino acid residues in regions 144 to 179, 188 to 245, and 219 to 245 of Php4 that could serve as NLSs. One of the characteristic features of a classical NLS is a degenerate consensus sequence of K(K/R)X(K/R) (where X indicates any amino acid residue) [6]. Analysis of Php4 using the NLS Mapper [34] prediction program highlighted three short regions containing positively charged residues that matched or partially matched the consensus K(K/R)X(K/R) motif. The first potential NLS, ^171^ KRIR ^174^ (amino acid residues 171–174) was found in region 144 to 179, whereas the second ^214^ KIRKR ^218^ (amino acid residues 214–218) and the third ^234^KSVKRVR ^240^ (amino acid residues 234–240) putative NLSs were located in region 188 to 245. In the case of region 219 to 245, it contained only the ^234^KSVKRVR ^240^ motif. To determine a functional NLS within Php4 that directs nuclear localization, we first mutated three positively charged amino acids, K^171^, R^172^, and R^174^ to Ala in full-length Php4 to generate Php4-N1. We also examined the effect of mutating K^214^, R^216^, K^217^, and R^218^ (Php4-N2) or K^234^, K^237^, R^238^, V^239^, and R^240^ (Php4-N3) to Ala on the ability of Php4 to localize to the nucleus (Fig. 3A). Results showed that Php4-N1, Php4-N2, and Php4-N3 mutants were efficiently targeted to the nucleus under iron starvation conditions, whereas their localization was predominantly cytoplasmic under high levels of iron (Fig. 3B). Iron-dependent nuclear-cytoplasmic trafficking of these mutants was similar to that of wild-type GFP-Php4 fusion protein (Fig. 3B). Subsequently, we combined the mutated residues within Php4-N3 with mutations in Php4-N2 (generating Php4-N4) or with mutations in Php4-N1 (generating Php4-N6) or with mutations in Php4-N1 and Php4-N2 (creating Php4-N7) (Fig. 3A). Similarly, mutated residues within Php4-N1 were combined with mutations in Php4-N2 to generate Php4-N5 mutant. *php4Δ* cells expressing Php4-N4 displayed nuclear accumulation following treatment with Dip. In contrast, Php4-N4 was exported out of the nucleus to the cytoplasm when cells had been treated with iron (Fig. 3B). Microscopy analysis showed that iron-starved cells expressing the *php4-N5* allele appeared to have less nuclear accumulation than wild-type protein or Php4-N1, -N2, -N3, and N4 mutants. On the other hand, cells harboring Php4-N6 and Php4-N7 did not show obvious nuclear accumulation under iron deprivation conditions (Fig. 3B). Under elevated iron levels, Php4-N5, Php4-N6, and Php4-N7 remained in the cytoplasm as observed in the case of wild-type GFP-Php4 protein. Taken together, these results revealed that Php4 harbors two functionally redundant NLSs, ^171^ KRIR ^174^ and ^234^ KSVKRVR ^240^, which could mediate nuclear import of Php4 independently.

**Figure 4:**
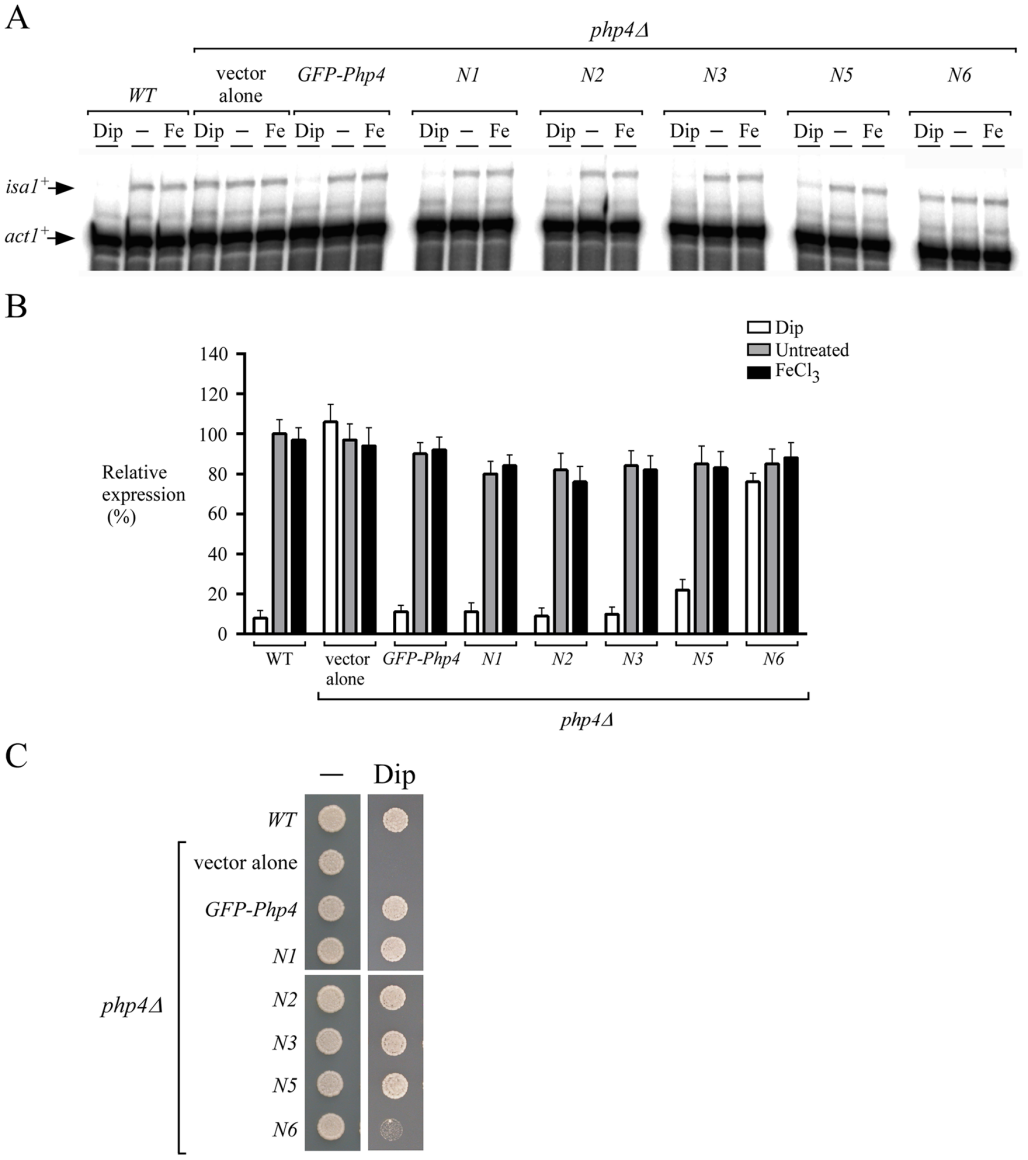
*Php4* NLSs are required for *Php4*-mediated repressive function. *A*, Cells carrying a disrupted *php4Δ* allele were transformed with an empty plasmid (vector alone) or plasmids expressing *GFP-php4^+^*, *GFP-php4^+^-N1*, *GFP-php4^+^-N2*, *GFP-php4^+^-N3*, *GFP-php4^+^-N5*, and *GFP-php4^+^-N6*. Transformed cells were grown under basal (−), iron-deficient conditions (250 μM Dip) or excess iron (100 μM FeCl_3_) (Fe). After total RNA extraction, *isa1^+^* and *act1^+^* steady-state mRNA levels were analyzed by RNase protection assays. Results shown are representative of three independent experiments. *B*, Quantification of *isa1^+^* levels after treatments shown in panel A. Data are shown as the mean of triplicate ± standard deviations. *C*, Wild-type (*WT*) and *php4Δ* cells expressing the indicated wild-type or mutant *GFP-php4* allele were spotted onto YES medium containing none (−) or 140 μM Dip and incubated at 30°C for 5 days. A *php4Δ* mutant containing an empty vector (vector alone) was used as a control strain known to be hypersensitive to Dip.

Because nuclear import is prerequisite to Php4 function, we hypothesized that mutations in Php4-N6 mutant (^171^ AAIA ^174^ and ^234^ ASVAAAA ^240^) would cause loss of Php4 function as well as produce cells defective in repression of the Php4 regulon in response to iron starvation. Indeed, cells expressing mutant *php4-N6* allele exhibited elevated *isa1^+^* mRNA levels that were virtually not repressed by iron starvation (Fig. 4A). In fact, steady-state levels of *isa1^+^* mRNA under low iron conditions were increased at least ∼7-fold above the levels of wild-type or a strain expressing a functional GFP-Php4 protein that was treated with Dip (Fig. 4B). In contrast, *isa1^+^* transcript levels were down-regulated under conditions of iron starvation in cells expressing the wild-type Php4 protein or Php4-N1, −N2, and -N3 mutants. In the case of the Php4-N5 mutant (^171^ AAIA ^174^ and ^214^ AIAAA ^218^), its mutations resulted in a ∼2-fold increase in the expression of the *isa1^+^* gene in the presence of low iron concentrations when compared to the levels observed in iron-starved cells expressing the wild-type GFP-Php4. Nonetheless, the levels of *isa1^+^* expression in the Php4-N5 mutant were still much lower under low iron than those under basal or iron-replete conditions (Fig. 4, A and B). Because the absence of Php4 led to a constitutive expression of iron-using genes, *php4Δ* mutant cells are known to be hypersensitive to low iron conditions (lack of optimization of iron utilization when iron is limited) (Fig. 4C). Results consistently showed that *php4Δ* cells expressing the *php4-N6* allele exhibited poor growth on low iron medium in comparison to wild-type cells (Fig. 4C). In contrast, cells expressing the wild-type GFP-Php4 protein or Php4-N1, -N2, -N3, and -N5 mutants were able to grow on medium containing Dip (Fig. 4C). Taken together, these results indicated that Php4 nuclear localization is necessary for Php4-mediated repressive transcriptional regulation of gene expression.

### Two NLSs trigger nuclear import by themselves

**Figure 5:**
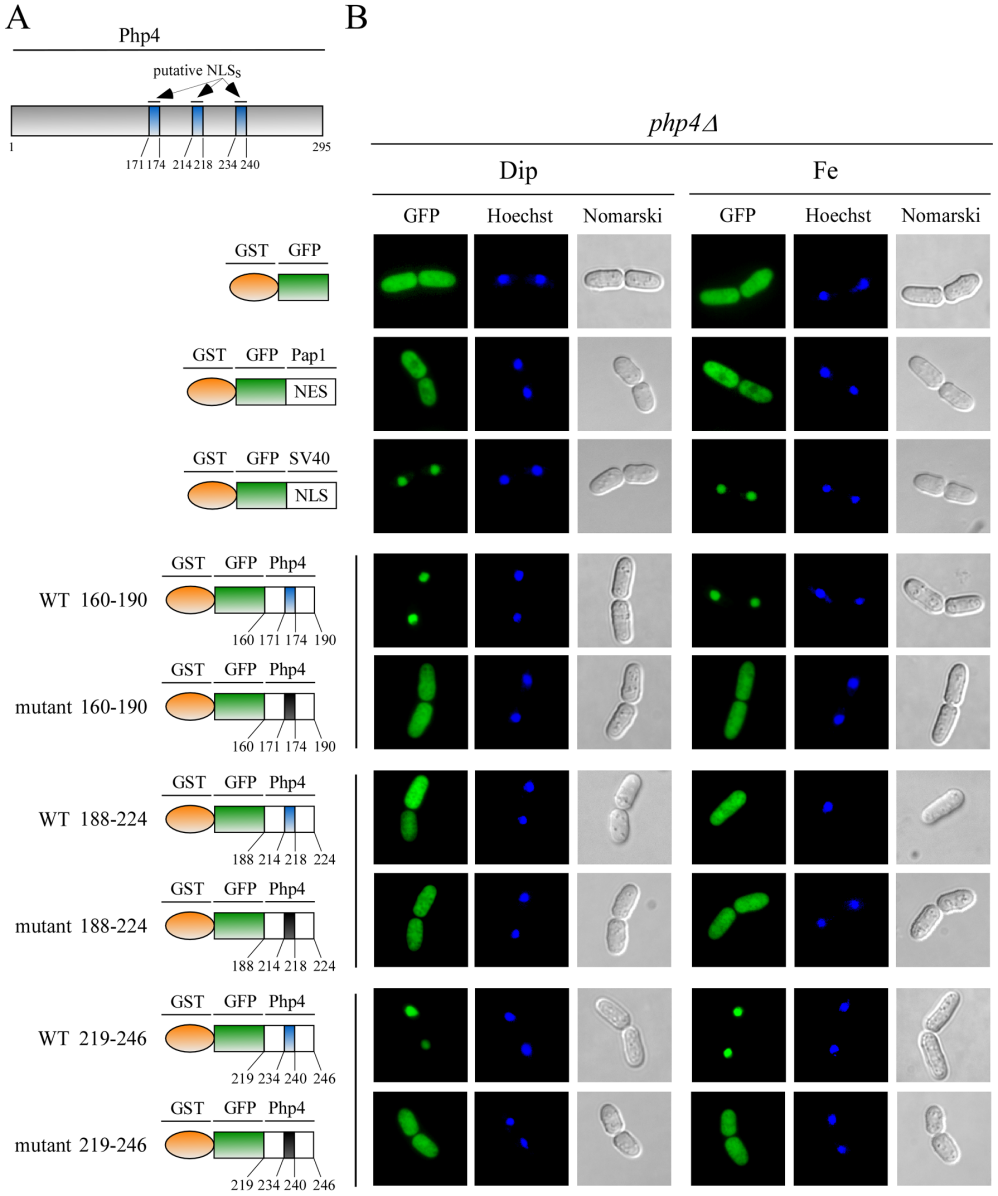
Amino acid fragments 160–190 and 219–246 of *Php4* contain nuclear import activity. *A*, Schematic representation of Php4 and several GST-GFP fusion reporter proteins containing NES or NLS regions of different proteins such as Pap1, SV40, and Php4. Color codes are, orange (GST), green (GFP), blue (putative Php4 NLS) and black (mutated NLS). *B*, Shown are representative *php4Δ* cells expressing GST-GFP, GST-GFP-Pap1NES, GST-GFP-SV40NLS, GST-GFP-Php4^160^NLS^190^, GST-GFP-Php4^160^mutantNLS^190^, GST-GFP-Php4^188^NLS^224^, GST-GFP-Php4^188^mutantNLS^224^, GST-GFP-Php4^219^NLS^246^, and GST-GFP-Php4^219^mutantNLS^246^, respectively. Cultures were grown in thiamine-free media for 12 h. After 3 h treatment in the presence of Dip (250 μM) or FeCl_3_ (Fe) (100 μM), cells were analyzed by fluorescence microscopy for GFP. As controls, nuclear DNA was visualized by Hoechst staining and cell morphology by Nomarski optics. The results shown are representative of five independent experiments.

To assess whether NLS regions of Php4 had the ability to trigger nuclear import, Php4 160–190, Php4 188–224, and Php4 219–246 fragments were fused to GST-GFP, which was used as a reporter protein in sufficiency experiments [35]. In addition, we examined the effect of mutating K^171^, R^172^, and R^174^ to Ala in Php4 160–190 (mutant 160–190), K^214^, R^216^, K^217^, and R^218^ to Ala in Php4 188–224 (mutant 188–224), and K^234^, K^237^, R^238^, V^239^, and R^240^ to Ala in Php4 219–246 (mutant 219–246) (Fig. 5A). *GST-GFP-Php4 160–190* (wild-type and mutant), *GST-GFP-Php4 188–224* (wild-type and mutant), and *GST-GFP-Php4 219–246* (wild-type and mutant) fusion alleles were expressed under the control of the thiamine-regulatable promoter [36]. This system allowed us to induce cellular pools of the above-mentioned fusion proteins and assess the effect of the presence of a given NLS (^171^KRIR^174^, ^214^KIRKR^218^, or ^234^KSVKRVR^240^) on their localization. Cells expressing GST-GFP-Php4 160-190 and GST-GFP-Php4 219–246 exhibited nuclear accumulation, whereas their mutant derivatives displayed a pancellular-fluorescence pattern in a manner similar to GST-GFP alone (Fig. 5B). In the case of GST-GFP-Php4 188–224, its location was cytoplasmic as well as nuclear, irrespective of the presence or absence of the basic residues K^214^, R^216^, K^217^, and R^218^ (Fig. 5B). Controls for nuclear import and export were GST-GFP-SV40NLS and GST-GFP-Pap1NES, respectively. Results showed that reporter proteins tested in sufficiency experiments were unaffected by cellular iron status (Fig. 5B). Furthermore, immunoblot analyses revealed that reporter proteins were stable and intact under the conditions analyzed (Figure S1 in File S1). Taken together, the data revealed that Php4 contains intrinsic determinants involved in nuclear import of the protein. Indeed, the Php4 160–190-(^171^KRIR^174^) and Php4 219–246-(^234^KSVKRVR^240^) regions function as transferable NLS sequence when fused with a reporter protein.

**Figure 6:**
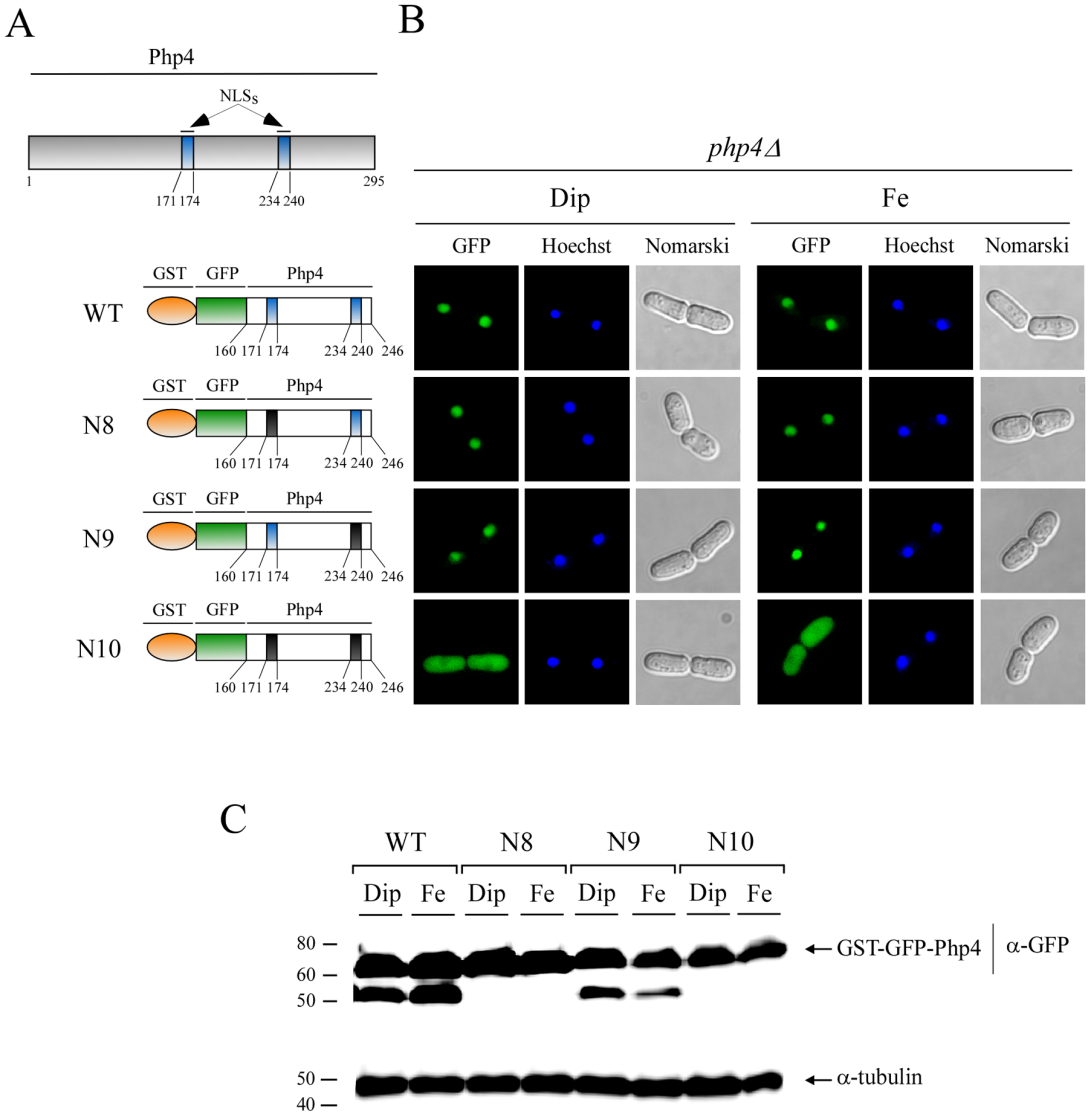
Identification of two functional *Php4* NLSs. *A,* Schematic representation of Php4 that shows relative locations of NLSs (blue boxes). The left bottom panel shows GST-GFP fusion proteins containing the amino acid fragment 160–246 of Php4, including wild-type (WT) and mutant (N8 to N10) versions. Color codes are, orange (GST), green (GFP), blue (NLS) and black (mutated NLS). Amino acid sequence numbers refer to the position relative to the first amino acid of Php4. *B*, Cells harboring a *php4Δ* deletion were transformed with the indicated integrative constructs. Cells were grown to early-logarithmic phase and then thiamine was withdrawn from cell cultures. Thiamine-free cultures were grown for 12 h, and then incubated in the presence of Dip (250 μM) or FeCl_3_ (Fe) (100 μM) for 3 h. Subsequently, cells were subjected to fluorescence microscopy for GFP detection. Cell morphology was examined through Nomarski optics (Nomarski) and nuclear DNA was detected by Hoechst staining. The results shown are representative of five independent experiments. *C*, Cell extracts were prepared from strains observed in panel B, and analyzed by immunoblotting. GST-GFP-^160^Php4^246^ (WT) and its mutant (N8 to N10) versions were detected using anti-GFP antibody. As an internal control, extracts preparations were probed with anti-α-tubulin antibody. The positions of the molecular weight standards are indicated to the left.

To further validate the observation that Php4 contained two functionally redundant NLSs, we expressed and analyzed a segment of Php4 comprising amino acid residues 160 to 246 using the GST-GFP reporter system (Fig. 6A). Amino acids K^171^, R^172^, and R^174^ were substituted by Ala in Php4 160–246 to generate Php4-N8. We converted the K^234^, K^237^, R^238^, V^239^, and R^240^ residues to Ala to generate Php4-N9. We also combined the mutated residues within Php4-N8 with those in Php4-N9 to generate the Php4-N10 mutant (Fig. 6A). Fluorescence microscopy analysis showed that *php4Δ* cells expressing mutant *GST-GFP-php4-N8* and *GST-GFP-php4-N9* alleles accumulated Php4 in the nucleus in a manner comparable to that of the wild-type GST-GFP-^160^Php4^246^ fusion protein (Fig. 6B). When both clusters of mutated residues were combined, GST-GFP-php4-N10 was not efficiently targeted to the nucleus, showing primarily cytosolic fluorescence and to less extent some pancellular distribution (Fig. 6B). Western blot analysis of cell extracts showed that the chimeric proteins were present at their expected size (Fig. 6C). Collectively, the results showed that the two NLS regions of Php4 (^171^KRIR^174^ and ^234^KSVKRVR^240^) are functionally redundant in the context of the truncated protein comprising amino acid residues 160 to 246. However, while the ^234^KSVKRVR^240^ element is fully functional in the truncated protein, this element is not fully competent to mediate nuclear import in the context of the full protein.

### Involvement of α- and β-karyopherins in import of Php4

**Figure 7:**
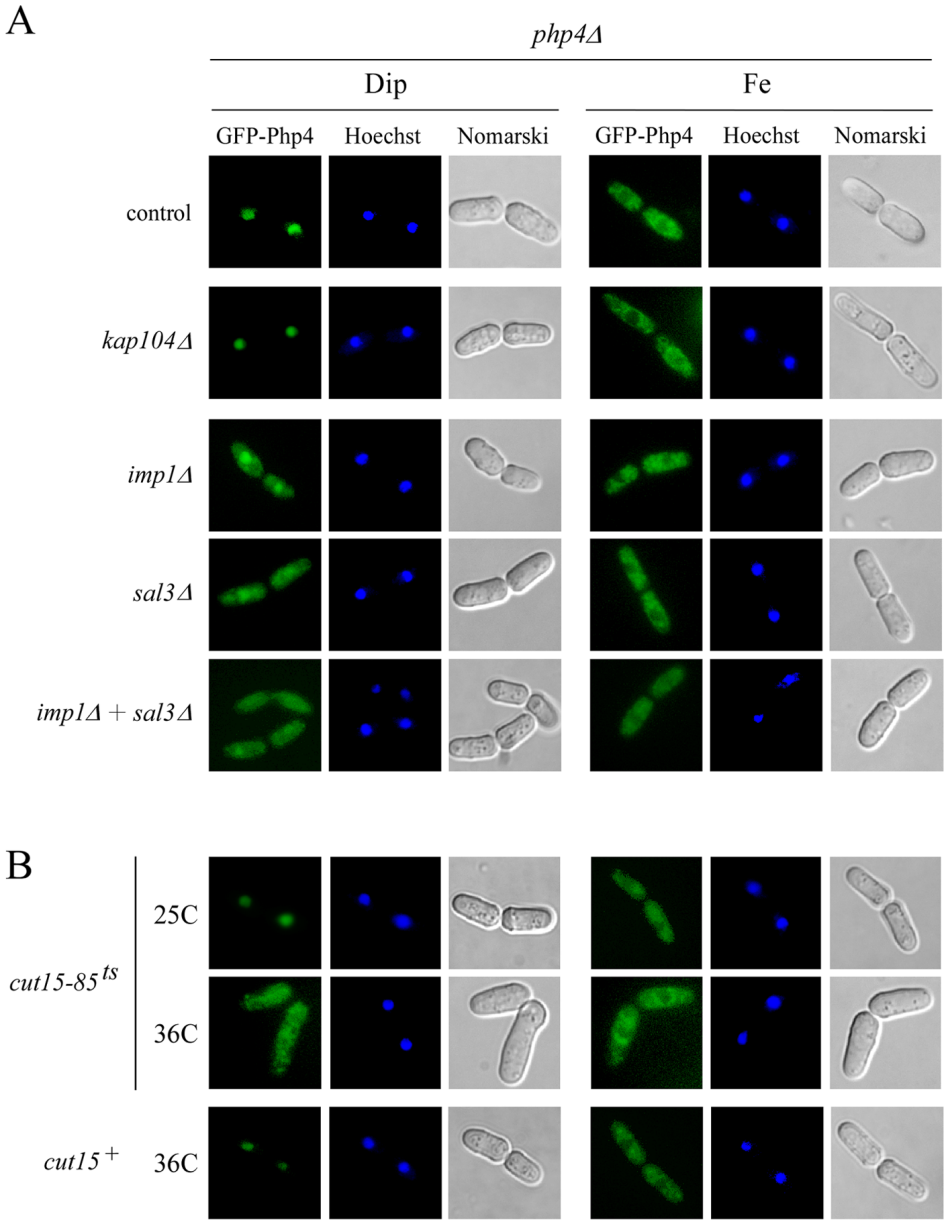
Inactivation of imp1^+^, cut15^+^ and sal3^+^ produced defect in nuclear import of GFP-*Php4*. *A,* An integrative plasmid expressing a functional GFP-tagged *php4^+^* allele was transformed into *php4Δ*, *php4Δ kap104Δ*, *php4Δ imp1Δ*, *php4Δ sal3Δ*, and *php4Δ sal3Δ imp1Δ* mutant strains. Mid-logarithmic phase cultures were treated with Dip (250 µM) or FeCl_3_ (Fe, 100 µM) for 3 h. Fluorescence microscopy was used to visualize cellular location of GFP-Php4. Cells were treated with Hoechst dye for nuclear DNA staining. Cell morphology was examined using Nomarski optics. *B,* Mid-logarithmic phase cultures of the indicated strains were grown at either the permissive (25°C) or nonpermissive (36°C) temperature for 1 h. Cultures were subsequently divided into four separate aliquots which were treated with Dip (250 µM) or FeCl_3_ (Fe, 100 µM) at permissive (25°C) or non-permissive (36°C) temperature. After 3 h treatment, cells were analyzed by fluorescence microscopy for GFP detection. The results shown are representative of five independent experiments.

Due to the fact that the two NLSs found in Php4 contained the degenerate consensus sequence of K(K/R)X(K/R), we concluded that both represented short basic classical NLSs [6]. To be transported in the nucleus, a protein containing a classical NLS is recognized by an importin α (karyopherin α or Kapα) protein, which serves as an adaptor. Subsequently, a karyopherin β1 (Kapβ1 or importin β1) binds the importin-α-cargo-complex to mediate its transport across the nuclear pore. Imp1 and Cut15 are the two importin α proteins in *S. pombe*. These two import adaptors have both unique and common binding cargoes [9]. To test whether the nuclear import of Php4 required Imp1, we disrupted the *imp1^+^* gene (*imp1Δ*) and determined the effect on the localization of GFP-Php4. Results showed that under conditions of iron starvation, the absence of Imp1 caused a partial mislocalization of GFP-Php4 to the cytoplasm, although a nuclear accumulation of GFP-Php4 was still observed to some extent (Fig. 7A). *cut15^+^* is essential for cell growth and our approach was to used *cut15–85* cells expressing a thermolabile Cut15 in which a *GFP-php4^+^* allele was previously integrated. At the permissive temperature (25°C) in which case Cut15 is functional, GFP-Php4 accumulated in the nuclei of iron-starved cells (Fig. 7B). However, incubation of iron-starved cells at the nonpermissive temperature (36°C) resulted in an alteration of GFP-Php4 nuclear localization and the GFP-Php4 signal was detected to both the cytoplasm and nucleus (Fig. 7B). Control experiments showed that GFP-Php4 was localized exclusively in the cytoplasm of wild-type and *cut15–85* strains when these transformed cells were incubated in the presence of iron under both temperature conditions (Fig. 7).

*S. cerevisiae* iron-responsive regulator Aft1 undergoes nucleo-cytoplasmic shuttling in response to changes in intracellular iron concentration in a manner analogous to Php4 [37], [38]. Aft1 accumulates in the nucleus upon iron starvation, whereas high iron concentrations result in nuclear export. Nuclear import of Aft1 is mediated by the Kapβ1 Pse1, which is a putative ortholog of *S. pombe* Sal3 [11], [38]. Based on this fact, we deleted the *sal3^+^* gene (*sal3Δ*). Results showed that disruption of Sal3 caused a partial mislocalization of GFP-Php4 to the cytoplasm under low levels of iron, suggesting that Sal3 also participated in nuclear import (Fig. 7A). As expected, when *sal3Δ* deletion cells were treated with iron, GFP-Php4 was primarily distributed in the cytoplasm (Fig. 7A). Nuclear accumulation of GFP-Php4 in response to iron starvation appeared to rely on more than one karyopherins. Thus, we investigated whether a *imp1Δ sal3Δ* double deletion would favor increased mislocalization of Php4 under low-iron conditions. Results showed that a double deletion of *imp1^+^* and *sal3^+^* exhibited a greater cytoplasmic accumulation of GFP-Php4, suggesting that Imp1 and Sal3 may use distinct nuclear import mechanisms for targeting Php4 to the nucleus (Fig. 7A). As a control, we tested whether the absence of Kap104 influenced Php4 localization. Kap104 is a Kapβ2 that specifically binds proline-tyrosine-NLS (PY-NLS) rather than classical NLS [12]. In this case, GFP-Php4 was properly localized in the nucleus in iron-starved *kap104Δ* cells, supporting the interpretation that the negative effect of the absence of Imp1, Cut15, or Sal3 on Php4 nuclear import was specific (Fig. 7A). When wild-type and mutant karyopherin strains were incubated in the presence of exogenous iron, GFP-Php4 was distributed in the cytoplasm of cells (Fig. 7). Taken together, the results revealed that Imp1, Cut15 or Sal3 could participate in nuclear accumulation of Php4 when cells are grown under low iron conditions.

**Figure 8:**
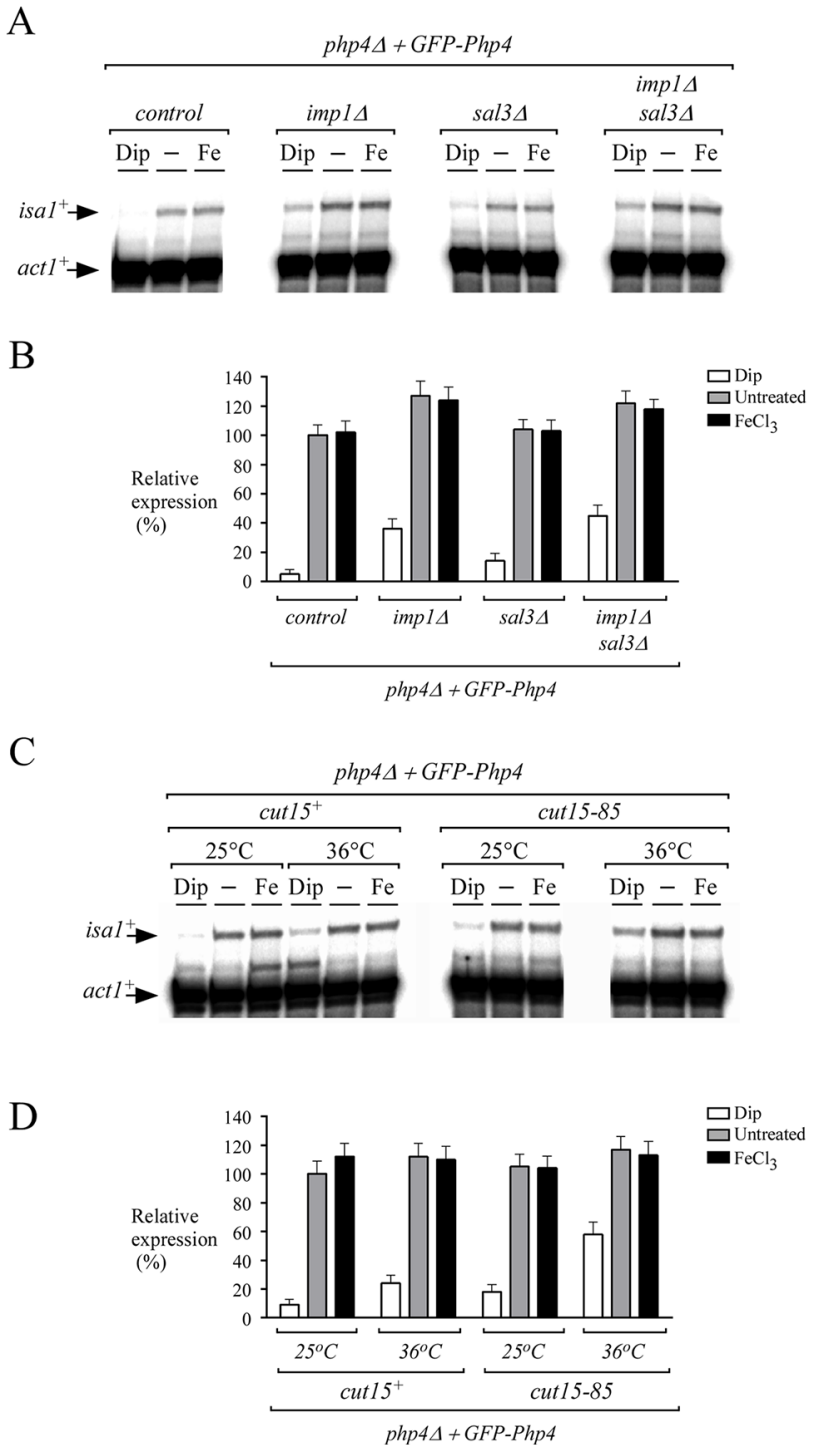
Loss of Imp1, Cut15 or Sal3 resulted in increased expression of isa1^+^ under low iron conditions. *A*, Strains harboring insertionally inactivated *php4Δ*, *php4Δ imp1Δ*, *php4Δ sal3Δ*, or *php4Δ imp1Δ sal3Δ* genes were transformed with the *GFP*-tagged *php4^+^* allele. The indicated strains were assessed for their ability to repress *isa1^+^* gene expression in the presence of Dip (250 µM) versus basal (−) or iron-replete (Fe, 100 µM) conditions. After 3 h of treatment, total RNA was prepared and then analyzed by RNase protection assays. Steady-state levels of *isa1^+^* and *act1^+^* mRNAs are shown with arrows. *B*, Quantification of three independent RNase protection assays, including the experiment shown in panel A. *C*, *php4Δ* and *php4Δ cut15–85* strains were transformed with an integrative plasmid expressing a functional GFP-Php4 protein. Mid-logarithmic phase cultures were divided into four aliquots which were treated with Dip (250 µM) or FeCl_3_ (100 µM) at permissive (25°C) or nonpermissive (36°C) temperature. After 3 h, total RNA was extracted and used in RNase protection protocol to determine *isa1^+^* and *act1^+^* mRNA levels. *D*, Quantification of *isa1^+^* transcript levels after treatments. Data are shown as the mean values of triplicate ± standard deviations.

Given the involvement of Imp1, Cut15 and Sal3 in nuclear import of Php4, we tested whether the repression of *isa1^+^* expression was affected in *imp1Δ*, *cut15-85* and *sal3Δ* mutant cells. Deletion of *imp1^+^* (*imp1Δ*) resulted in steady-state levels of *isa1^+^* that were increased (∼30%) in cells treated with Dip in comparison with iron-starved control cells (Fig. 8). In the case of disruption of *sal3^+^* (*sal3Δ*) that resulted in a modest upregulation (∼10%) of *isa1^+^* transcription under low iron conditions. Similarly to *imp1Δ* cells, mRNA levels of *isa1^+^* were upregulated (∼40%) in *imp1Δ sal3Δ* cells, especially in the case of iron-starved control cells (Fig. 8). Similar increases in *isa1^+^* mRNA levels were observed in *imp1Δ*, *sal3Δ* and *imp1Δ sal3Δ* cells expressing an endogenous Php4 protein (Figure S2 in File S1). We also examined steady-state mRNA levels of *isa1^+^* in *cut15–85* cells expressing a thermolabile Cut15. *php4Δ* and *php4Δ cut15–85* cells expressing GFP-Php4 were grown at the permissive temperature (25°C). At mid-logarithmic phase, cells were divided in aliquots which were then incubated at permissive (25°C) or nonpermissive (36°C) temperature in the presence of Dip (250 μM), FeCl_3_ (100 μM), or left without treatment. At 25°C, a temperature where Cut15 was functional, cells displayed very low *isa1^+^* transcript levels under low iron conditions (Dip). In contrast, *isa1^+^* mRNA levels were up-regulated under basal and iron-replete conditions (Fig. 8). At nonpermissive temperature (36°C), inactivation of Cut15 resulted in a 34% increase in *isa1^+^* transcription under low iron conditions compared to levels of *isa1^+^* observed in a *cut15^+^* strain under the same conditions (Fig. 8). In *cut15–85* cells expressing an endogenous Php4 protein, inactivation of Cut15 resulted in a 20% increase in *isa1^+^* transcription under iron starvation conditions (Figure S2 in File S1). As expected, *isa1^+^* mRNA levels in both untreated and iron-treated *php4Δ cut15–85 GFP-php4^+^* or *cut15–85* cells were induced as compared to iron-starved cells (Fig. 8 and Figure S2 in File S1). Collectively, these results indicated that Php4 is less competent to repress gene expression under low iron conditions in the absence of Imp1, Cut15 or Sal3.

### Imp1, Cut15 and Sal3 are interacting partners of Php4

**Figure 9:**
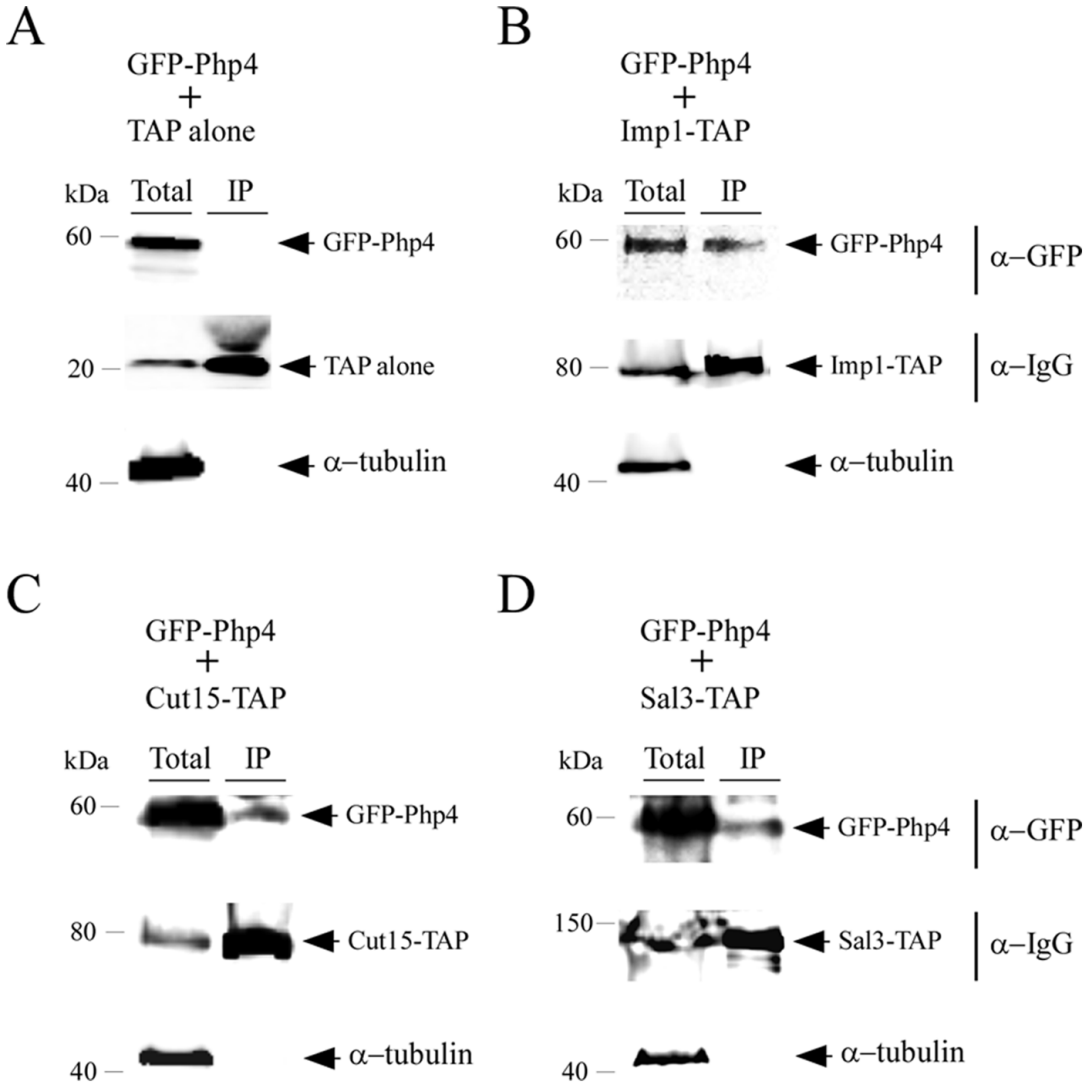
*Php4* interacts with Imp1, Cut15, and Sal3 in S. pombe. *php4Δ* cells expressing GFP-tagged Php4 and TAP alone (*A*), GFP-tagged Php4 and TAP-tagged Imp1 (*B*), GFP-tagged Php4 and TAP-tagged Sal3 (*C*), or GFP-tagged Php4 and TAP-tagged Cut15 (*D*) were grown to mid-logarithmic phase in EMM without thiamine in the presence of Dip (250 µM). Extracts (Total) were subjected to immunoprecipitation (IP) using IgG-Sepharose beads. The bound proteins were eluted and analyzed by immunoblot assays using a mouse anti-GFP antibody (α-GFP). A portion of the total cell extracts (∼2%) was included to ascertain the presence of proteins prior to chromatography. As additional controls, aliquots of whole-cell extracts and bound fractions were probed with an anti-mouse IgG antibody (α-IgG) and an anti-tubulin antibody (α-tubulin). The positions of the molecular weight of protein standards (in kDa) are indicated on the left-hand side.

Given the fact that inactivation of *imp1^+^*, *cut15^+^* or *sal3^+^* negatively altered import of Php4 to a different extent, we examined whether Php4 could form complexes with Imp1, Cut15 or Sal3 *in vivo*. To address this possibility, we investigated Php4 capacity to interact with these proteins using TAP pull-down experiments. In these assays, we used iron-starved cells co-expressing distinct pairs of fusion proteins, including GFP-Php4 and Imp1-TAP, GFP-Php4 and Cut15-TAP, GFP-Php4 and Sal3-TAP or GFP-Php4 and TAP (Fig. 9). Total cell extracts were incubated in the presence of IgG-Sepharose beads that selectively bound unfused TAP or TAP-tagged proteins. This strategy allowed an enrichment of Imp1, Cut15 or Sal3 and detection of their potential interacting partners. Western blot analysis of proteins retained by the beads using an anti-GFP antibody revealed that GFP-Php4 was present in the immunoprecipitate fraction of cells expressing Imp1-TAP, Cut15-TAP or Sal3-TAP (Fig. 9). In contrast, GFP-Php4 was absent in the bound fraction of cells expressing TAP alone (Fig. 9). Whole-cell extract fractionation was confirmed using an antibody directed against α-tubulin. Results showed that α-tubulin was present in total cell extracts but not in the retained protein fractions (Fig. 9). To ascertain the steady-state protein levels of Imp1-TAP, Cut15-TAP, or Sal3-TAP, Western blot analyses of both whole cell protein preparations and bound fractions were performed using an anti-IgG antibody (Fig. 9). Taken together, these results showed the existence of Php4-Imp1, Php4-Cut15 and Php4-Sal3 interactions in *S. pombe*.

## Discussion

Php4-like proteins are widely distributed among fungal species [20], [39]. These proteins include Hap43 (from *Candida albicans*), AnHapX (from *Aspergillus nidulans*), AfHapX (from *Aspergillus fumigatus*) and CnHapX (from *Cryptococcus neoformans*) [40]–[43] [44]. Of note, *Saccharomyces* species are one of the rare groups that lack Php4 orthologs. Although Php4-like proteins are key nuclear regulators for preventing futile expression of genes encoding iron-using proteins under low-iron conditions, the nature of their NLSs and the mechanisms responsible for triggering their nuclear import have remained poorly characterized. In this study, we have identified two functionally independent and redundant NLSs that are responsible for delivery of Php4 into the nucleus. The first NLS (^171^KRIR^174^) possessed a sequence that matched the degenerate consensus K(K/R)X(K/R) motif, which represents one of the two types of conventional monopartite NLSs. Furthermore, classical monopartite NLSs are known to specifically bind Kap α proteins. The second NLS (^234^KSVKRVR^240^) is a modified version of the first one. It has ^237^KRVR^240^ [K(K/R)X(K/R)] as a basic core motif and few flanking residues (^234^KSV^236^) immediately upstream of the core basic residues. These properties represent a modified pattern of classical monopartite NLS that has been previously shown to be competent for binding with Kap α proteins [27]. Indeed, a previous study has shown that the RVSKRPR motif, which is highly reminiscent to KSVKRVR found in Php4, is specifically recognized by Kap α [27]. When we examined the effect of mutating ^234^K to Ala on the ability of GST-GFP-^219^Php4^246^ protein to localize to the nucleus, we observed only a weak mislocalization of the protein to the cytoplasm (in comparison with an unmodified GST-GFP-^219^Php4^246^). Yet, most GST-GFP-^219^Php4^246^ ^234^K→A signal was detected in the nucleus in response to iron starvation (unpublished data). When ^237^KRVR^240^ were mutated to Ala residues in GST-GFP-^219^Php4^246^, the mutant exhibited a pancellular distribution pattern, revealing that the basic core amino acid residues were essential for nuclear import (unpublished data).

Consistent with the amino acid composition of the two Php4 NLSs, we found that the two *S. pombe* Kap α proteins, Imp1 and Cut15, were involved in nuclear import of Php4. This observation meant that Php4 is a common cargo for Imp1 and Cut15. This situation has been reported before. SV40 NLS is functional in *S. pombe* and has been used to assess nuclear protein import competence. As observed in the case of Php4, both *cut15-85* and *imp1Δ* mutant cells were less efficient at accumulating a SV40 NLS fusion protein in the nucleus than wild type cells [9], revealing that Imp1 and Cut15 have overlapping functions for the import of an SV40 NLS-containing protein. Similarly to Php4, it has been reported that *S. pombe* transcription factor Pap1 interacts with both Imp1 and Cut15 [9]. Neither *imp1Δ* nor *cut15–85* mutant cells were competent to efficiently import Pap1 into the nucleus as compared to wild-type cells. This observation suggested an overlapping function of Imp1 and Cut15 for nuclear import of Pap1. In *S. cerevisiae*, Kap95 is a Kapβ1 involved in the nuclear import of proteins with classical NLSs. One pathway by which Kap95 mediates nuclear import of cargo proteins involves its association with a Kap α protein. One could envision that *S. pombe* Kap95, which is essential for cell viability, is required for the Imp1- or Cut15-mediated nuclear import of Php4. However, the potential involvement of Kap95 remains speculative at this time and needs further investigation.

In general, protein containing NLSs that are recognized by Kap α proteins are transported as a trimeric complex with Kapβ1 proteins. However, it has been shown in the case of some proteins that their nuclear import can be mediated by distinct Kaps or groups of Kaps. These proteins include histones, ribosomal proteins and stress-responsive transcription factors such as Asr1 and AlcR [45], [46]. These findings led us to examine whether some nonessential members of the Kap β family could be required for nuclear import of Php4. Results showed that the inactivation of Sal3 caused a mislocalization of Php4 to the cytoplasm (although a significant proportion of Php4 could still be seen into the nucleus). In *S. pombe*, Sal3 is the ortholog of *S. cerevisiae* Pse1. Interestingly, Pse1 is required for the nuclear localization of the iron-responsive transcription factor Aft1 in *S. cerevisiae*. Although Aft1 is a transcriptional activator and in contrast, Php4 is a transcriptional repressor, both are active and accumulate in the nucleus under conditions of iron starvation. Similarly to Php4, Aft1 possesses two functionally independent NLSs. Although their amino acid composition (KPKKKR and RKPK) is different than those of Php4 (KRIR and KSVKRVR), each of these NLSs is monopartite and is closely related to the consensus sequence K(K/R)X(K/R). However, as opposed to Kap α proteins that are required for nuclear import of Php4, *S. cerevisiae* Kap α (Srp1) is not involved in nuclear import of Aft1. Furthermore, it has been shown that nuclear translocation of Aft1 is exclusively dependent on Pse1 in *S. cerevisiae* and does not depend on other Kap β family members [38].

In contrast, some proteins in *S. cerevisiae* are import substrates of more than one Kaps. For instance, Kap114, Kap95, Kap123, Pse1, and Kap104 recognize NLSs present in histones H2A and H2B, whereas these Kaps mediate nuclear transport of Asr1 [46]. Based on these data, it is likely that *S. pombe* Php4 interacts with more than one type of nucleo-cytoplasmic factors, thereby leaving more options for its nuclear import when iron levels are low. However, the question whether one Php4 NLS is more specific than the others in being recognized by either Kaps α (Imp1 and Cut15) or Kap β1 (Sal3) awaits further studies.

In *A. nidulans*, the CCAAT-binding factor is composed of the HapB, HapC, HapE and HapX subunits [40], [47]. Whereas HapC and HapE lack NLSs, HapB contains one functional NLS. In the case of HapX, the presence of functional NLS has not been reported. Under iron sufficient conditions, while the *HAPX* gene is repressed, HapB, HapC and HapE are expressed and assembled as a heterotrimeric complex. To enable cells to provide equimolar concentrations of HapB/C/E subunits to the nucleus, HapB subunit acts as a primary cargo for nuclear import of HapC and HapE. According to a proposed model, HapC and HapE have first to form a heterodimer that is transported into the nucleus only in complex with HapB by way of a piggy-back mechanism [47]. Although the nuclear import mechanism of *S. pombe* CCAAT-binding Php2/3/5 subunits is unknown, we investigated whether nuclear import of Php4 was dependent of the presence of these subunits. Under iron-limiting conditions, disruption of *php2^+^*, *php3^+^* and *php5^+^* had no effect on the nuclear import of Php4. Results showed that Php4 accumulated within the nucleus of iron-starved *php2Δ php3Δ php5Δ* triple mutant cells. In the presence of iron, Php4 exhibited a steady-state distribution in the cytoplasm of both *php2^+^*/*3^+^*/*5^+^* and *php2Δ/3Δ/5Δ* strains. We concluded that nucleocytoplasmic trafficking of Php4 was Php2/3/5-independent. This mechanism is different in comparison with the piggy-back nuclear import mechanism that occurs for the heterotrimeric CCAAT-binding complex in *A. nidulans*.

Our findings suggest that NLS-mediated import of Php4 is not iron-regulated, as we found that the presence of iron did not affect the nuclear localization of the three GST-GFP-Php4 NLS fusion proteins (GST-GFP-^160^Php4^190^, GST-GFP-^219^Php4^246^, and GST-GFP-^160^Php4^246^) (Figs 5 and 6). Furthermore, in the context of full-length protein, when nuclear export sequence (NES) of Php4 was mutated, Php4 exhibited a constitutive nuclear localization under both iron-depleted and iron-replete conditions [21]. This observation suggested that the recognition of Php4 NLSs by Imp1, Cut15 or Sal3 occurred regardless of iron conditions. Nevertheless, it is intriguing to note that Php4 NLSs (positions 171 to 174 and 234 to 240) are included in a region of Php4 from residues 152 to 254 that is known to be required for interaction with the GRX domain of Grx4 [22]. As opposed to the TRX domain, the GRX domain of Grx4 interacts in an iron-dependent manner with Php4. Under high-iron conditions, the GRX domain interacts with the region 152 to 254 of Php4, which may induce conformational changes that negatively affect interactions between NLSs and their import receptors. This may contribute in cytoplasmic accumulation of Php4 under iron-replete conditions. In contrast, under iron-limiting conditions, the GRX domain is no longer able to interact with Php4, which may favor associations between Php4 NLSs and Kaps, therefore contributing in nuclear accumulation of Php4. Although this dynamic interplay may occur in the context of the full-length Php4 protein, further investigation is needed to address this possibility.

## Supporting Information

File S1 Figure S1, Detection of intact GST-GFP and GST-GFP fusion proteins. Figure S2, Inactivation of *imp1Δ*, *cut15–5* or *sal3Δ* resulted in increased expression of isa1^+^ under iron starvation conditions.
(DOC)
