# Supplementary material for: Characterization of the Nuclear Import Mechanism of the CCAAT-Regulatory Subunit Php4
Source: PLoS One. 2014 Oct 17;9(10):e110721. doi: 10.1371/journal.pone.0110721 (PMC4201560; doi:10.1371/journal.pone.0110721)
Supplement: File S1 — Figure S1, Detection of intact GST-GFP and GST-GFP fusion proteins. Figure S2, Inactivation of imp1Δ, cut15–5 or sal3Δ resulted in increased expression of isa1+ under iron starvation conditions. (DOC) [file pone.0110721.s001.doc]

**SUPPLEMENTAL DATA**

Khan *et al.*

**Figure S1.** *Detection of intact GST-GFP and GST-GFP fusion proteins*.

**
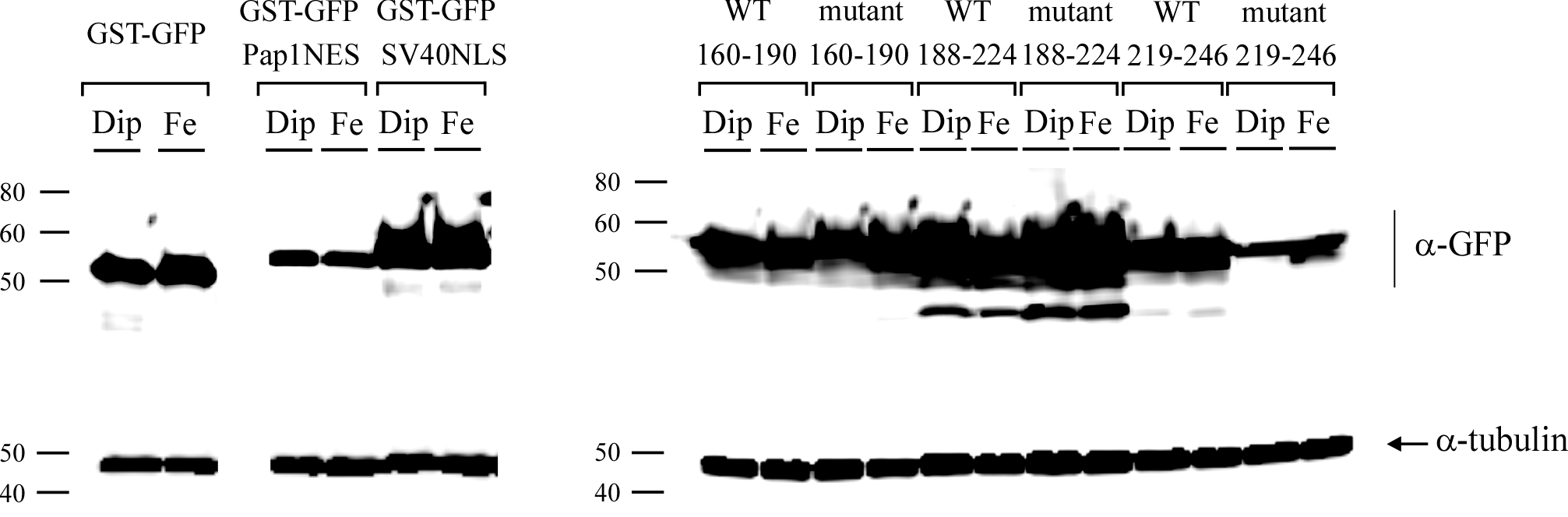
**

**Figure S1.** *Detection of intact GST-GFP and GST-GFP fusion proteins*. Cell lysates from aliquots of the cultures described in figure 5 were analyzed by immunoblotting using either anti-GFP or anti-α-tubulin (as an internal control) antibody. The positions of the molecular weight of protein standards (in kDa) are indicated on the left-hand side.

**Figure S2.** *Inactivation of imp1Δ, cut15-85 or sal3Δ resulted in increased expression of isa1^+^ under iron starvation conditions.*

**
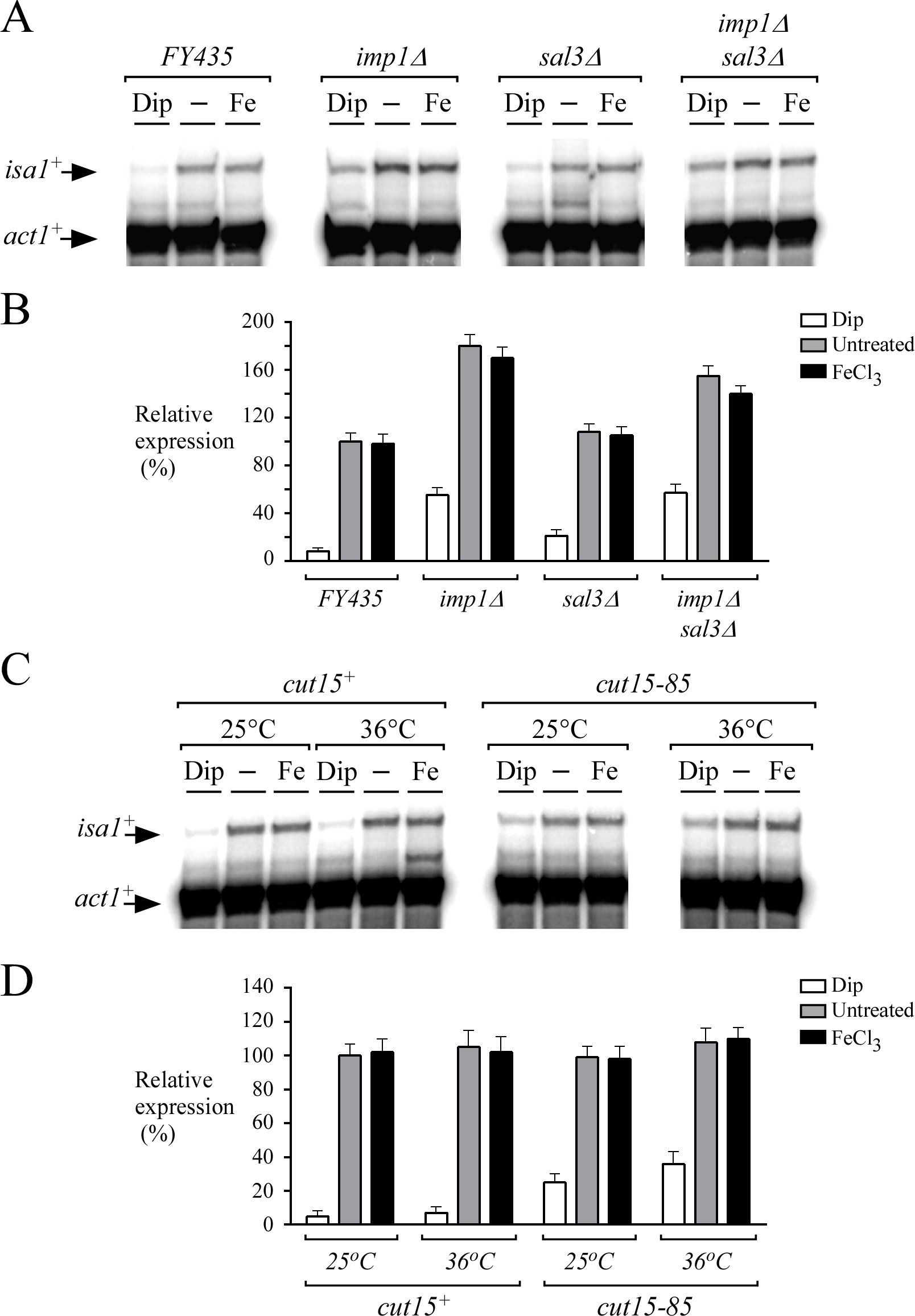
**

**Figure S2.** *Inactivation of imp1Δ, cut15-85 or sal3Δ resulted in increased expression of isa1^+^ under iron starvation conditions. A*, The indicated strains containing an endogenous Php4 were assessed for their ability to repress *isa1^+^* gene expression in the presence of Dip (250 µM) versus basal (-) or iron-replete (Fe, 100 µM) conditions. After 90 min of treatment, total RNA was prepared and then analyzed by RNase protection assays. Steady-state levels of *isa1^+^* and *act1^+^* mRNAs are shown with arrows. *B*, Quantification of three independent RNase protection assays, including the experiment shown in panel A. *C*, *cut15^+^* and *cut15-85* strains expressing an endogenous Php4 were grown to mid-logarithmic phase and then were divided into four aliquots which were treated with Dip (250 µM) or FeCl_3_ (100 µM) at permissive (25^o^C) or nonpermissive (36^o^C) temperature. After 3 h, total RNA was extracted and used in RNase protection protocol to determine *isa1^+^* and *act1^+^* mRNA levels. When indicated (-), cells were left untreated. *D*, Quantification of *isa1^+^* transcript levels after treatments. Data are shown as the mean values of triplicate ± standard deviations.
